# P-108. Ibuzatrelvir Potently Reduced Viral RNA Levels Despite the High Rate of Anti-S Seropositivity: a Post Hoc Analysis of Serology in the Phase 2b Study in Adults Without Risk Factors for Severe COVID-19

**DOI:** 10.1093/ofid/ofaf695.336

**Published:** 2026-01-11

**Authors:** Jin Hyang Kim, Alex Knutson, Justin Smith, Shunjie Guan, Luke Chen, Mahta Mortezavi, Abigail Sloan, Anindita Banerjee, Mary Lynn Baniecki, Craig Hyde, Charlotte Allerton, Negar Niki Alami

**Affiliations:** Pfizer, Lake Forest, IL; Pfizer, Lake Forest, IL; Pfizer, Lake Forest, IL; Pfizer, Lake Forest, IL; Pfizer, Lake Forest, IL; Pfizer, Lake Forest, IL; Pfizer, Lake Forest, IL; Pfizer, Lake Forest, IL; Pfizer Inc, Cambridge, Massachusetts; Pfizer, Lake Forest, IL; Pfizer, Lake Forest, IL; Pfizer Inc., Chicago, Illinois

## Abstract

**Background:**

We previously reported that ibuzatrelvir 600mg, an oral SARS-CoV-2 M^pro^ inhibitor, demonstrated significantly greater reduction from baseline (BL) in viral RNA levels (viral load [VL]) compared with placebo at day 5 (1.2 log_10_ copies/ml, 80% CI -1.5, -0.8) among adults with baseline VL ≥4 log_10_ copies/ml. In this report, we evaluated the impact of pre-existing neutralizing antibody (nAb) levels on viral clearance, where nearly all participants had positive anti-Spike (anti-S) antibodies, indicating prior COVID-19 vaccination or infection, similar to the real world.

**Figure:**
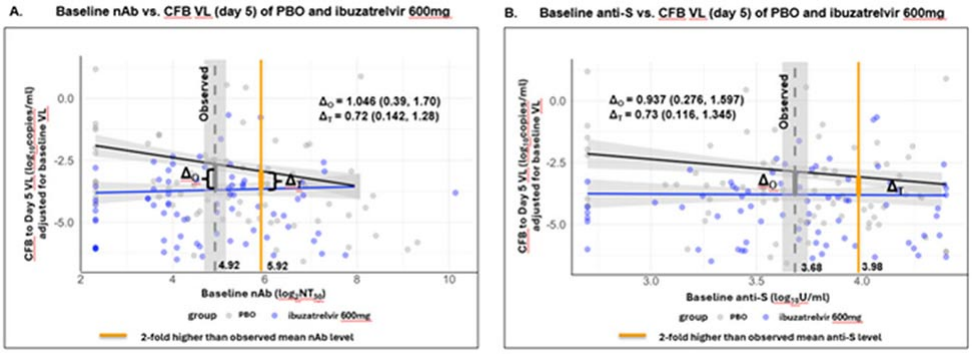

**Methods:**

This is a post hoc exploratory analysis of the phase 2b study evaluating virologic response and safety of ibuzatrelvir in adults without risk factors for severe COVID-19 during Omicron era. Participants were randomized 1:1:2:2 to receive oral ibuzatrelvir 100mg, 300mg, or 600mg or placebo twice daily for 5 days. Nasopharyngeal SARS-CoV-2 VL at BL and day 5, and serum anti-S and nAb (against XBB.1.5) levels at BL and day 10 were analyzed. The relationship between BL Ab (nAb and anti-S) levels and day 5 VL reduction by treatment group was evaluated using linear regression. The fitted mean difference in day 5 VL reduction between the ibuzatrelvir 600mg and placebo groups was estimated at a BL Ab level 2-fold higher than the observed mean Ab level.

**Results:**

The mean BL anti-S level was 3.67 log_10_ U/ml with 99.6% anti-S positivity, while the mean nAb level was 5.06 log_2_ NT_50_ with 17% of participants below detectable levels. BL anti-S levels were significantly (r=0.65, p< 0.001) correlated with BL nAb levels. Higher BL nAb levels were significantly (p=0.004) associated with greater VL reduction at day 5 in placebo, but not in the ibuzatrelvir-treated group. Modeling of these study data projected that even at a mean BL nAb level 2-fold higher than was observed, VL reduction by ibuzatrelvir 600mg would have been approximately 5-fold (0.72 log_10_ copies/ml, 95% CI: 0.14, 1.29) greater than placebo (Figure A). Modeling of anti-S yielded a similar estimate (0.73 log_10_ copies/ml, 95% CI: 0.12, 1.35) (Figure B).

**Conclusion:**

VL reduction by ibuzatrelvir was observed in the phase 2b study despite evolving immunity, and the modeling suggests that this will also occur even at higher mean Ab levels than were observed in this study.

**Disclosures:**

Jin Hyang Kim, PhD. RPh, Pfizer: employee|Pfizer: Stocks/Bonds (Public Company) Alex Knutson, Ph.D., Pfizer: employee|Pfizer: Stocks/Bonds (Public Company) Justin Smith, M.S., Pfizer: employee|Pfizer: Stocks/Bonds (Public Company) Shunjie Guan, PhD in Statistics, Pfizer: employee|Pfizer: Stocks/Bonds (Public Company) Luke Chen, MBBS, Pfizer: employee|Pfizer: Stocks/Bonds (Public Company) Mahta Mortezavi, MD, Pfizer: employee|Pfizer: Stocks/Bonds (Public Company) Abigail Sloan, Ph.D., Pfizer: employee|Pfizer: Stocks/Bonds (Public Company) Anindita Banerjee, Ph.D., Pfizer: employee|Pfizer: Stocks/Bonds (Public Company) Mary Lynn Baniecki, PhD, Pfizer, Inc: Salaried Employee|Pfizer, Inc: Stocks/Bonds (Public Company) Craig Hyde, Ph.D., Pfizer: employee|Pfizer: Stocks/Bonds (Public Company) Charlotte Allerton, PhD, Pfizer: employee|Pfizer: Stocks/Bonds (Public Company) Negar Niki Alami, MD, Pfizer: Employee|Pfizer: Stocks/Bonds (Public Company)

